# Primary and subsequent secondary aorto-enteric fistulae in the setting of chronic Q fever

**DOI:** 10.1093/jscr/rjac579

**Published:** 2023-01-28

**Authors:** Dana Tabbara, Adam Frankel, Iain Thomson

**Affiliations:** Department of Surgery, Princess Alexandra Hospital, Woolloongabba, QLD, Australia; Department of Surgery, Princess Alexandra Hospital, Woolloongabba, QLD, Australia; Faculty of Medicine, University of Queensland, Brisbane, QLD, Australia; Department of Surgery, Princess Alexandra Hospital, Woolloongabba, QLD, Australia; Faculty of Medicine, University of Queensland, Brisbane, QLD, Australia

**Keywords:** aorto-enteric fistula, Q fever, EVAR

## Abstract

We report the case of an 80-year-old male with stage three kidney disease, who survived a primary aorto-enteric fistula (AEF) in the setting of chronic Q fever after presenting with melena and syncope. His initial surgical treatment included endovascular aortic repair. Type 2 endoleak was present post-operatively. Six months later, he was diagnosed with a secondary AEF after syncope and large volume hematemesis. He was definitively treated with an open explant of his stent, repair of the duodenum and bilateral axillofemoral bypass. Two years later, he remains active and independent on life-long antibiotics.

## INTRODUCTION

Primary aorto-enteric fistula (AEF) describes a direct communication between the native aorta and a part of the gastrointestinal tract, most commonly the duodenum [1]. Secondary AEF occurs in the presence of a graft repair of an abdominal aortic aneurysm. Though rare, AEF is associated with a mortality rate of up to 50%, owing to the rapid exsanguination caused by the aortic defect [2].

To our knowledge, Arima *et al.* [3] describe the only other case of secondary AEF occurring after primary AEF. In this analogous case, an endovascular approach was used to manage the primary fistula. Secondary AEF occurred in the setting of persistent type 2 endoleak and an enlarged aneurysmal sac, with the explanted graft not yielding any organisms after culture [3]. We acknowledge the high chance of reporting bias but suggest that these two cases should prompt caution regarding the use of EVAR in the definitive management of AEF, as ongoing infection at the repair site is likely to lead to re-fistulisation [1, 3–5].

## CASE REPORT

An 80-year-old retired male farmer was brought by ambulance to an outer metropolitan hospital with melaena and syncope. His past medical history included stage 3 chronic kidney disease (eGFR 40 ml/min/1.73m^2^) but no risk factors for gastrointestinal bleeding.

**Figure 1:**
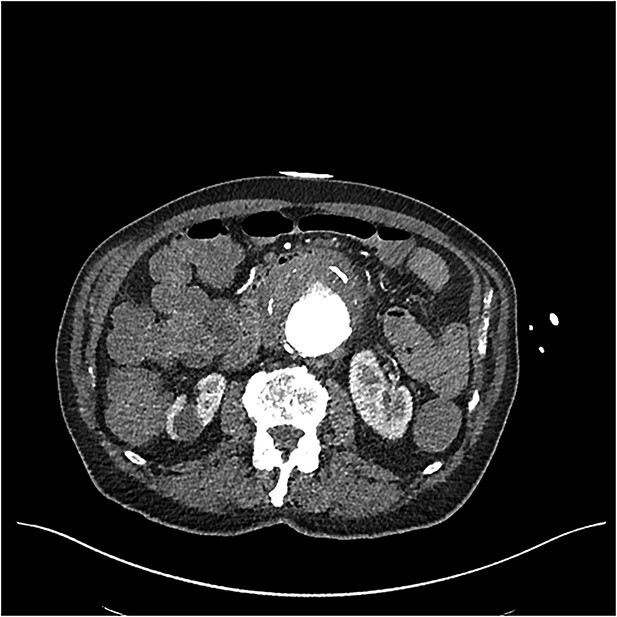
CT angiogram on initial presentation demonstrating an aneurysmal abdominal aorta but no contrast extravasation.

CT showed a likely AEF associated with a 7 cm inflamed aneurysmal sac posterior to the third part of the duodenum (Fig. 1). While no active bleeding was seen, large clots were present in the stomach and colon. Goal-directed resuscitation was commenced and he was transferred to a tertiary centre. After general and vascular surgical review, the patient proceeded to theatre for urgent endovascular aneurysm repair (EVAR) and duodenal repair.

**Figure 2:**
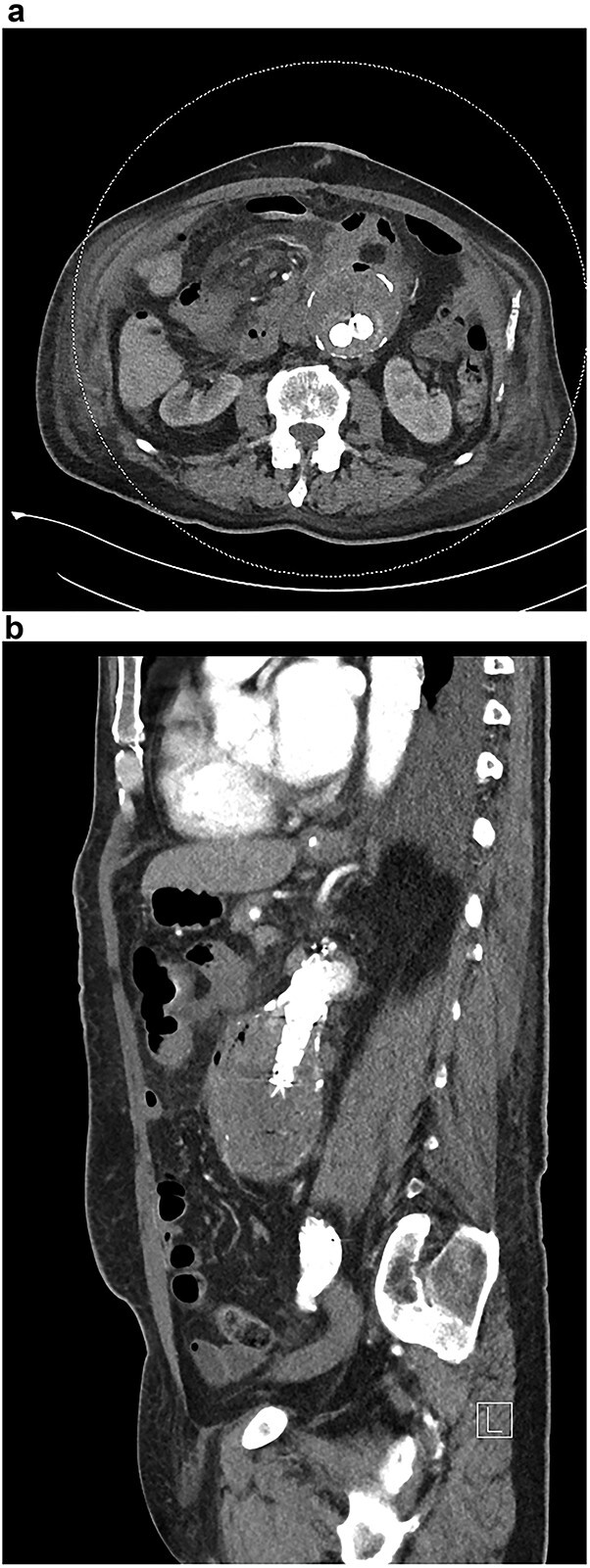
(**a** and **b**) CT angiogram after EVAR demonstrating endoleak.

**Figure 3:**
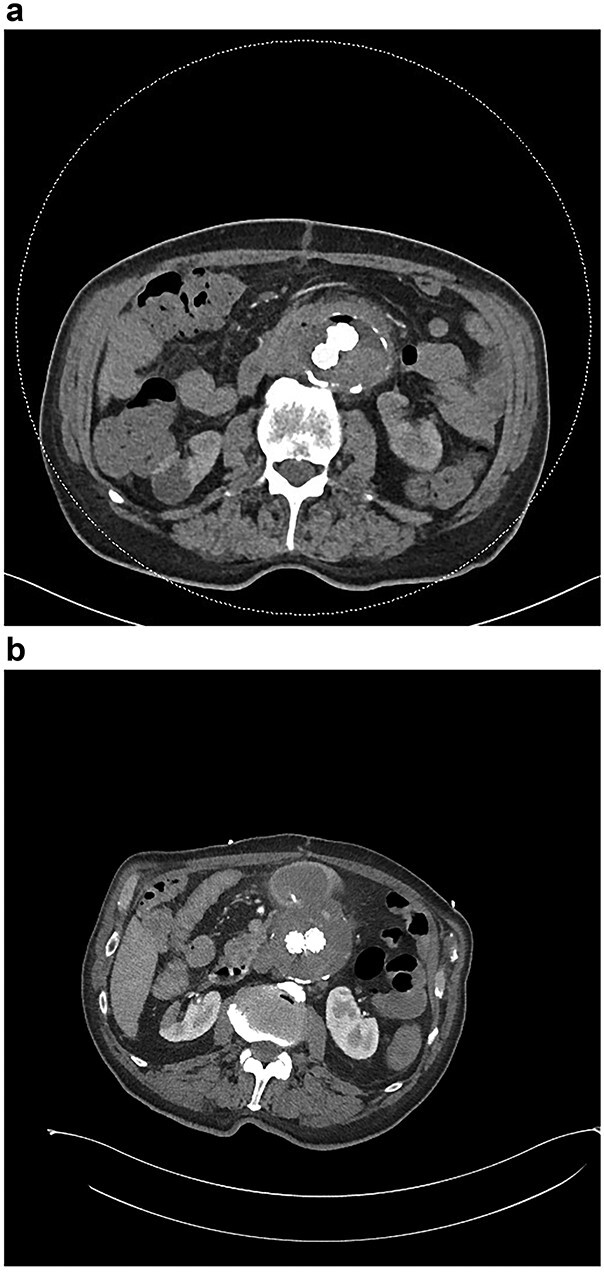
(**a**) CT angiogram demonstrating new gas in the aneurysmal sac 6 weeks post-repair; (**b**) CT angiogram just prior to the second repair, demonstrating ongoing endoleak and continuity of the duodenum and sac; part of the duodenal clip is visible.

A stent with iliac limb extensions was deployed. EndoAnchors (Medtronic) were applied proximally. Final angiogram demonstrated patent renal and iliac arteries but a type 2 endoleak (Fig. 2a and b). The duodenum was accessed via midline laparotomy, with *a* < 1 cm defect in the posterior wall of the third part of the duodenum closed in two layers. A pedicled omental patch was interposed. Intra-operative samples grew *Streptococcus gordonii* and *Actinomyces odontolyticus*, species typically found in the oral cavity. Given the prior occupational history of the patient, Q fever serology was also requested and antibodies to the phase 1 antigen returned strongly positive, suggestive of chronic Q fever. After specialty infectious diseases input, an 18-month course of doxycycline and hydroxychloroquine and a life-long course of amoxicillin-clavulanate were commenced. He suffered post-operative delirium. On post-operative Day 10, a small amount of blood in the nasogastric tube prompted an urgent oesophagogastroduodenoscopy. The repair site had some minor ooze and was clipped. Repeat endoscopy 1 month later identified a small mucosal defect in D3 and an over-the-scope clip was applied. Six weeks after repair, outpatient blood tests demonstrated a rising white cell count. CT angiography demonstrated new gas in the aneurysmal sac (Fig. 3a).

Six months after the index presentation, a secondary AEF was diagnosed. The patient presented similarly with syncope after massive hematemesis and a 5-day history of melena. CT angiography again demonstrated the type 2 endoleak, with active contrast extravasation into the third part of the duodenum (Fig. 3b). Once resuscitated, the patient was treated with an open explant of the stent, repair of the duodenum and bilateral axillofemoral bypass. Residual anchors from the previous EVAR remained *in situ*. Over two years later, he remains active and independent despite his incisional hernia, playing golf multiple times per week. His eGFR is 34 ml/minute/1.73m^2^. He remains on long-term anti-microbial suppression.

## DISCUSSION

The contribution of persistent type 2 endoleak to the development of secondary AEF is unclear in our case as the aneurysmal sac shrunk with time. More likely, the graft was subject to prolonged inflammation in the setting of chronic Q fever, a recognised cause of vascular lesions [5, 6]. Unfortunately, the graft explant was not sent for culture and we cannot exclude the contribution of odontogenic and other bacteria to re-fistulisation. The process of fistulisation is complex, and no definitive infectious cause may be found even after culture [3, 4].

Our patient had multiple risk factors for graft infection and re-fistulisation including his underlying chronic kidney disease, advanced age and emergent endovascular repair [7]. Indeed, many consider EVAR to be a bridging procedure prior to more definitive open or extra-anatomical approaches to repair [3, 4]. Therefore, in addition to long-term antimicrobial therapy, in appropriate candidates, we suggest consideration of early elective re-operative management, ideally extra-anatomical reconstruction with explant of the EVAR graft, to reduce the risk of recurrent AEF.

## CONFLICT OF INTEREST STATEMENT

No conflict of interest to declare.

## FUNDING

None.

## References

[1] Chung J . Management of aortoenteric fistula. Adv Surg 2018;52:155–77. 30098611 10.1016/j.yasu.2018.03.007

[2] Chopra A , Cieciura L, Modrall JG, Valentine RJ, Chung J. Twenty-year experience with aorto-enteric fistula repair: gastrointestinal complications predict mortality. J Am Coll Surg 2017;225:9–18. 28161484 10.1016/j.jamcollsurg.2017.01.050

[3] Arima D , Suematsu Y, Kurahashi K, Shimizu T, Nishi S, Yoshimoto A. Recurrence of aortoenteric fistula after endovascular aortic repair. Ann Vasc Dis 2020;13:90–2. 32273930 10.3400/avd.cr.19-00106 PMC7140165

[4] Antoniou GA , Koutsias S, Antoniou SA, Georgiakakis A, Lazarides MK, Giannoukas AD. Outcome after endovascular stent graft repair of aortoenteric fistula: a systematic review. J Vasc Surg 2009;49:782–9. 19028054 10.1016/j.jvs.2008.08.068

[5] Wegdam-Blans MC , Vainas T, van Sambeek MR, Cuypers PW, Tjhie HT, van Straten AH, et al. Vascular complications of Q-fever infections. Eur J Vasc Endovasc Surg 2011;42:384–92. 21622013 10.1016/j.ejvs.2011.04.013

[6] Karhof S , van Roeden SE, Oosterheert JJ, Bleeker-Rovers CP, Renders NHM, de Borst GJ, et al. Primary and secondary arterial fistulas during chronic Q fever. J Vasc Surg 2018;68:1906–13.e1. 29685511 10.1016/j.jvs.2018.01.044

[7] Antonello RM , D'Oria M, Cavallaro M, Dore F, Cova MA, Ricciardi MC, et al. Management of abdominal aortic prosthetic graft and endograft infections. A multidisciplinary update. J Infect Chemother 2019;25:669–80. 31182331 10.1016/j.jiac.2019.05.013

